# Influence of the patient-practitioner interaction context on acupuncture outcomes in functional dyspepsia: study protocol for a multicenter randomized controlled trial

**DOI:** 10.1186/s12906-017-1869-y

**Published:** 2017-07-14

**Authors:** Seok-Jae Ko, Jae-Woo Park, Jungtae Leem, Ted J. Kaptchuk, Vitaly Napadow, Braden Kuo, Jessica Gerber, Laurie Dimisko, Inkwon Yeo, Junhee Lee, Jinsung Kim

**Affiliations:** 1 Department of Gastroenterology, College of Korean Medicine, Kyung Hee University, 26 Kyung Heedae-ro, Dongdaemun-gu, Seoul, 02447 Republic of Korea; 2 DongShin Korean Medicine Hospital, 351, Omok-ro, Yangcheon-gu, Seoul, 07999 Republic of Korea; 3 Program in Placebo Studies, Beth Israel Deaconess Medical Center, Harvard Medical School, Boston, MA 02215 USA; 4 Martinos Center for Biomedical Imaging, Department of Radiology, Massachusetts General Hospital, Medical School. Boston, Harvard, MA USA; 5 Gastroenterology Unit, Massachusetts General Hospital, Harvard Medical School, Blake 4, 55 Fruit St, Boston, MA 02114 USA; 6 BioMEMS Resource Center, Massachusetts General Hospital, Harvard Medical School, Boston, MA USA; 7 Department of Statistics, Sookmyung Women’s University, 100 Cheongpa-ro 47-gil, Yongsan-gu, Seoul, 04310 Republic of Korea; 8 Department of Sasang Constitutional Medicine, College of Korean Medicine, Kyung Hee University, 26 Kyungheedae-ro, Dongdaemun-gu, Seoul, 02447 Republic of Korea

**Keywords:** Functional dyspepsia, Acupuncture, Randomized controlled trial, Augmented interaction, Limited interaction

## Abstract

**Background:**

In the treatment of functional dyspepsia, the placebo effect has been reported to be high, and the influence of the patient-practitioner relationship may be a major component of this effect. The specific and non-specific effects of acupuncture cannot be easily distinguished, and the patient-practitioner relationship may influence the total therapeutic effect in clinical practice. There have been no studies that investigate the influence of patient-practitioner relationship on acupuncture treatment for patients with functional dyspepsia.

**Methods:**

Patients with postprandial distress syndrome, a functional dyspepsia subtype, will be recruited at three hospitals (two in Korea and one in USA) for an international, multi-center, randomized, patient/assessor-blinded, clinical trial. The total anticipated sample size is 88. The participants will be randomly allocated into two groups: an augmented interaction group and a limited interaction group. Acupuncture, with total 12 acupoints, will be performed twice weekly for 4 weeks in both groups. Trained practitioners will provide an “augmented” or “limited” interaction context, as determined by random allocation. The primary outcome measure is the proportion of responders, the proportion of participants who answer “yes” to more than half of the adequate relief questions during the study. Secondary outcome measures include questionnaires for quality of life and symptoms of dyspepsia, and maximum tolerable volume of nutrient drink test. Data will be collected at baseline and following 4 weeks of acupuncture.

**Discussion:**

This study will evaluate the influence of the patient-practitioner interaction on clinical effects of acupuncture in patients with functional dyspepsia.

**Trial registration:**

CRIS Identifier: (KCT0002229).

## Background

Functional dyspepsia (FD) describes various upper abdominal symptoms, including abdominal pain, fullness, early satiety, and epigastric burning, without organic disease [1]. There is no cure for FD, and the therapeutic approach is complicated by the diversity of symptoms, and the inconsistency between symptoms and pathophysiology. While medication can be helpful in the short term, medication results in a temporary reduction of symptoms [2]. As a result, patients with FD are turning to alternative and complementary medicine, such as acupuncture [3].

The placebo effect is a non-specific improvement that occurs unrelated to the specific therapeutic regimen. Such non-specific clinical benefits can be divided into three components: the patient’s response to observation and assessment (Hawthorne effect); the patient’s response to the administration of a therapeutic regimen (placebo effect); and the patient’s response to the patient-practitioner relationship [4–6]. In patients with functional gastrointestinal disorders, up to 50% of the therapeutic effect may be due to the placebo effect [7, 8]. A study of patients with irritable bowel syndrome showed that non-specific effects could produce statistically and clinically significant outcomes, and that the patient-practitioner relationship was the most important component [9]. In patients with gastroesophageal reflux disease, an expanded patient-practitioner visit resulted in greater improvement of symptoms, when compared to a standard empathic medical visit. Complementary and integrative medicine consultations may enhance the placebo effect [10].

Specific effect and non-specific effects cannot be easily distinguished in complementary and integrative medicine. The influence of the patient-practitioner relationship could be a meaningful component of therapeutic experience. Therefore, to enhance the effect of treatment in a clinical setting, understanding the patient-practitioner relationship is required. To date, there have been no studies investigating the influence of patient-practitioner relationship on acupuncture treatment for patients with functional dyspepsia.

This study is designed to investigate the cognitive, emotional, and symbolic contextual factors embedded in the patient-practitioner relationship. These factors are assumed to modulate, and potentially amplify, the effect of acupuncture. Contextual factors include the use of emphatic attention, natural expressions of warmth and support, positive expectations, attentive listening, thoughtful silence, and appropriate touch. We believe that a patient-practitioner relationship “augmented” with positive components will more intensely modulate emotion, cognition, and reward when compared to “limited” context treatments.

Acupuncture is a suitable intervention to determine whether an expanded patient-practitioner interaction affects medical outcomes. The therapeutic encounter with acupuncture is “holistic,” since it includes the physical, mental/emotional, and spiritual dimensions of the individual. Therefore, acupuncture provides a suitable framework for an enhanced interaction. The acupuncture assessment interview can be adapted to extensively engage in physical, emotional, cognitive, existential, and social discourse, and can provide the patient with a sense of being cared for and understood.

The goal of this study is to investigate the influence of the patient-practitioner relationship on the effect of acupuncture on symptoms of patients with functional dyspepsia. We will answer the questions:

1. What is the role for acupuncture in functional dyspepsia?
2. What are the underlying mechanisms of acupuncture’s holistic clinical encounter? We will also compare the non-specific effects between Asian patients, who are likely familiar with acupuncture, and Western patients, through studies in both Asia and USA.

## Methods

### Design

**Fig. 1:**
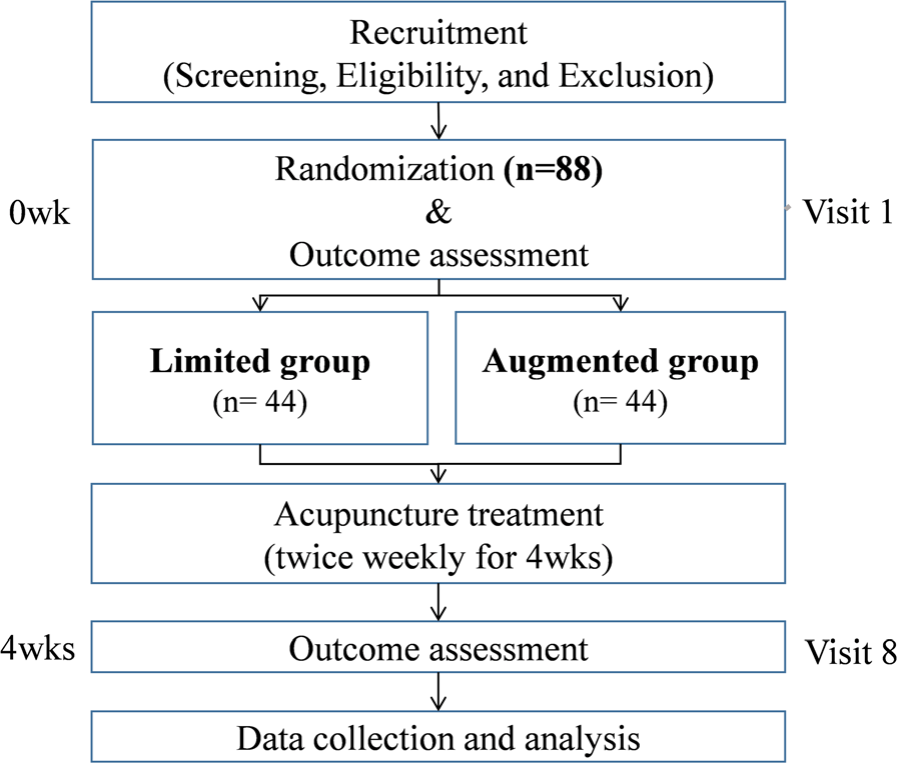
Flow chart of the study

This study is planned as a multicenter, randomized controlled, patient/assessor-blinded, clinical trial to investigate the effect of two different patient-practitioner interactions on symptoms, and quality of life, of patients with functional dyspepsia. This study will be conducted in Kyung Hee University Hospital at Gangdong, Kyung Hee University Medical Center (Seoul, Republic of Korea), and the Massachusetts General Hospital (Boston, MA, USA), from January 2017 to May 2018. Participants will be randomized in a 1:1 fashion to receive either an augmented or limited medical visit. All participants will be treated for functional dyspepsia by acupuncture for 4 weeks (8 sessions). Symptoms and quality of life will be measured by questionnaires. The practitioner will discuss patient problems, including dyspeptic symptoms, at each visit in either the augmented or limited format. The initial visit will be video recorded after obtaining the participants’ consent. The Clinical Trial Institutional Review Board (IRB) of Kyung Hee University Hospital at Gangdong (No. KHNMCOH 2016–04–007-001), Kyung Hee University Medical Center (No. KOMCIRB – 150,914 – HR-040), and Massachusetts General Hospital (No. 2016P001981/PHS) have approved the study. The study will be carried out in accordance with the standards of the International Committee on Harmonization on Good Clinical Practice, and the revised version of the Declaration of Helsinki. This study has been registered under the identifier No. KCT0002229 at CRIS (http://cris.nih.go.kr). Figure 1 provides a study flow chart.

### Participants

A total of 88 patients will be recruited from three centers (Kyung Hee University Hospital at Gangdong, 29 patients; Kyung Hee University Medical Center, 30 patients; and Massachusetts General Hospital, 29 patients).

### Inclusion and exclusion criteria

#### Inclusion criteria

Patients who meet all of the following criteria will be considered for enrollment. The inclusion criteria are:

1. Age between 20 and 65 years.
2. Meeting the definition of functional dyspepsia, and postprandial distress syndrome, as a subtype of FD, according to the Rome III criteria [1].
3. A degree of dyspepsia more than 40 points on the visual analogue scale (0, no symptoms; 100, the most severe symptoms).
4. Individuals who agreed not to begin new treatments associated with FD during the study. Participants may continue FD prescriptions, over the counter or alternative medications, and any psychological treatments, as long as they have been on a stable regimen for 30 days prior to the start of the study. Participants also agree not to change medications or dosage during the study.
5. Voluntarily agreed and sign an informed consent form.

#### Exclusion criteria

Exclusion criteria include:

1. Recent abnormal esophagogastroduodenoscopy or evidence of any organic diseases affecting gastrointestinal tract (e.g. erosive esophagitis, peptic ulcer, dysplasia, mucosa-associated, lymphoid tissue lymphoma, esophageal cancer, or gastric cancer).
2. Signs of irritable bowel syndrome.
3. Serious symptoms, including weight loss, black or tarry stool, or dysphagia.
4. Cardiac, pulmonary, hepatic or renal disease, or mental illness.
5. Gastrointestinal surgery, except for appendectomy performed more than 6 monthsprior to the study.
6. Women who are pregnant or breastfeeding.
7. Individuals taking drugs that could affect the gastrointestinal tract. A minimum washout period of 2 weeks is required before participating in the trial.
8. Participation in another clinical trial within 1 month of this trial.
9. Positive for human immunodeficiency virus infection.
10. Individuals taking herbal medicine or an oriental remedy for dyspepsia, or drugs that are considered inappropriate for this study.
11. Difficulties in attending the trial (e.g., paralysis, serious mental illness, dementia, drug addiction, time constraints, severe disorders of vision or hearing, illiteracy, etc.). Patients who have severe depression (more than 24 points in Beck Depression Inventory) will be excluded [11].
12. Other issues that could interfere with acupuncture treatment (e.g., clotting disorders, leukopenia, pacemaker, epilepsy, or anticoagulant therapy).

### Recruitment procedures

Participants will be recruited in hospital outpatient clinics; by banner advertisements placed on the notice boards in the local communities; advertisements in the local newspaper, bus, and subway stations; and advertisements on web sites visited by patients with digestive diseases. Patients interested in participating will contact the Clinical Research Coordinator for further information. Patients will be screened for eligibility by a telephone interview and scheduled for an initial visit for serologic test including white blood cell, hemoglobin, hematocrit, platelet, aspartate transaminase, alanine transaminase, blood urea nitrogen, and creatinine test. After being fully informed about the procedures and risks, and given sufficient time to make a decision, participants who are deemed eligible will provide written, informed consent. Participants have the right to withdraw from the trial at any time during the study period.

### Randomization, allocation concealment, and blinding

After participants have completed a baseline evaluation, they will be randomized to two groups: the augmented group and the limited group. The randomization will be carried out using random number lists, created in accordance with the PROC PLAN of SAS 9.4 (SAS Institute Inc., Cary, NC, USA) by an independent statistician. The acupuncture practitioner will be unaware of the allocation until opening an opaque, sealed envelope. Participants will not be informed that we are studying the patient-practitioner interaction. They will be told the study is "Acupuncture for the treatment of functional dyspepsia: An international multicenter trial." Participants will be informed of the purpose of the study in a debriefing session at the end of the study. The Clinical Research Coordinator evaluating the variables will be blinded to the allocation.

### Intervention

#### Augmented group

The augmented group intervention contains two of components. First, a holistic assessment based on Traditional Korean Medicine in which the practitioner will discuss physical, psycho-emotional, social, and existential/spiritual parameters. Second, specific context enhancements, such as warmth and support, empathic attention, attentive listening, thoughtful silence, shared explanation and exploration of meaning, positive expectation, and therapeutic touch, including pulse and tongue evaluation. Many of these context enhancements are included in acupuncture practitioners’ style of treatment.

In the augmented interaction group, in addition to questions about FD symptoms, the practitioner will explore the emotional experience of FD, and the meaning of FD as a condition experienced by the patient. As part of the interview process, the practitioner will engage in the seven specific enhancements. The use of these specific enhancements will occur at different times, in as natural a way as possible.

#### Limited group

The limited group will be conducted like a serious scientific encounter. It is meant to provide limited patient-practitioner interaction, and to evaluate the impact of the intervention in isolation from other context effects. Palpation of the pulse and abdominal examination will not be done. Practitioners will not explore the emotional experience, or the meaning of the illness. Furthermore, practitioners will not engage in any of the context enhancements, but will engage the patient as naturally as possible with a “matter of fact” and “to-the-point” demeanor.

The practitioner will read a script to the patient explaining that the patient-practitioner interaction must to be kept to a minimum because of the scientific nature of a randomized controlled trial. Subsequent treatment sessions will be performed within the limits defined in the first session, thus the context will remain restricted. The acupuncture treatment will take 20 min and will involve no additional communication between practitioner and patient.

While maintaining a personable and reassuring demeanor, the practitioner will avoid engaging in education or reframing of the participant’s pain or condition, and will avoid any verbal interaction, other than that necessary to evaluate the participant’s initial condition, change, and progress. The practitioner will strive to maintain a natural and comfortable interaction with the patient, as to not compromise the validity of the treatment. The differences between the augmented and limited groups are detailed in Table 1.

**Table 1:** Guideline for augmented and limited interaction context

Augmented Interaction context	Limited Interaction context
1. Warm, Friendly Style	1. Neutral, Impersonal Style
- Shake hands - Thank patient for participation in study - Frequent use of patient’s name. - Sit face to face, not separated by a desk - Appropriate smiling - Appropriate use of humor	- Do not initiate hand shaking - Do not thank patient for participation - Infrequent use of patient’s name - Sit separated by desk - Limit smiling - Avoid humor
2. Attentive	2. Distracted
- Frequent eye contact - Leans in at appropriate points - Appropriate use of head nods - Limited use of computer or clipboard	- Infrequent eye contact - Bodily posture conveys distance - Minimal use of head nods - Frequent use of computer or clipboard
3. Active Listening	3. Minimal Active Listening
- Open-ended questions that emphasize patient’s experience of illness - Avoid interrupting patient - Patient speaks more than clinician - Allow some silence for contemplation - Reflections - Clarifications	- Closed-ended questions (yes or no) - Tightly focused on review of systems - Interrupt patient to keep to script - Clinician does most of the talking - Fill silences quickly - Avoid reflections - Avoid clarifications
4. Personalized Content	4. Generic Content
- The clinician will encourage the patient to speak for the majority of the time - The clinician’s explanation of the illness and treatment will be personalized, using the patient’s own words wherever possible	- The clinician will dominate the interview, speaking for the majority of the time - The clinician’s explanation of the illness and treatment will be generic with no personalization for the patient
5. Empathic Connection	5. Reduced Empathy and Connection
- Partnership (We’ll work together) - Empathy (That must have been painful) - Validate emotions	- No expression of partnership - No empathy expression (but not callousness) - Avoid emotional content
6. Mirroring	6. No Mirroring
- Mirror patient’s prosody (volume, pace, intonation) - Avoid medical jargon - Use vocabulary familiar to patient	- No effort at mirroring patient’s prosody - Use medical jargon - No effort to use vocabulary familiar to patient

#### Treatment fidelity

In order to evaluate compliance with the protocol and treatment fidelity, the first acupuncture session for all subjects will be videotaped. Ten percent (10%) of videotapes will be randomly reviewed to assure that the practitioners in the augmented and limited groups follow the standard operating procedure. Patients will be made aware of the recording as part of the informed consent process, and only the interview will be recorded. Participants will be told that the purpose of the recording is to assure practitioners compliance with the protocol. Videotaping of treatment will not be performed, and the practitioner will assure that the video is turned off when the acupuncture treatment starts.

#### Acupuncture treatment

Acupuncture will be applied in the same manner in both groups. A total of 12 acupoints (Hegu [LI 4], Zusanli [ST 36], Taichong [LR 3], Quze [PC 3], Gongsun [SP 4] and Liangqiu [ST 34] bilaterally) will be used twice weekly for 4 week (8 sessions). Acupuncture will be performed as follows. The acupuncture needle will be applied to the participant’s skin. Then, after turning on the timer, the acupuncture needles will be rotated right and left for 3 s at each acupoint (total 12 acupoints). After 20 min, the timer is turned off, and the acupuncture needles will be rotated right and left for another 3 s at each acupoint, and then the acupuncture needles will be removed.

Detailed information regarding the acupuncture rationale is described in the revised STandards for Reporting Interventions in Clinical Trials of Acupuncture (STRICTA) [12] shown in Table 2.

**Table 2:** Acupuncture treatment details based on the STRICTA (Macpherson H et al., [12]) checklist

Item	Detail
1. Acupuncture rationale	1a) Style of acupuncture - Manual acupuncture based on traditional meridian theory.
	1b) Reasoning for treatment provided, based on historical context, literature sources, and/or consensus methods, with references where appropriate - Based on the traditional meridian theory, clinical experience, and consensus by the experts in acupuncture and FD.
	1c) Extent to which treatment was varied - No additional acupoints allowed.
2. Details of needling	2a) Number of needle insertions per subject per session (mean and range where relevant) - Fixed 12 acupoints.
	2b) Names (or location if no standard name) of points used (uni/bilateral) - LI 4, ST36, LR3, PC3, SP4 and ST34 (bilateral)
	2c) Depth of insertion, based on a specified unit of measurement, or on a particular tissue level - From 5 to 30 mm.
	2d) Response sought (e.g. de qi or muscle twitch response) - ‘De qi’ sensation
	2e) Needle stimulation (e.g. manual, electrical) - Manual acupuncture
	2f) Needle retention time - Twenty minutes.
	2g) Needle type (diameter, length, and manufacturer or material) - A sterilized stainless steel needle (0.25 × 40 mm, DONGBANG ACUPUNCTURE INC., Ungcheon, Boryeong, Korea).
3. Treatment regimen	3a) Number of treatment sessions - Eight sessions.
	3b) Frequency and duration of treatment sessions - Twice weekly for 4 weeks, 20 min for each session.
4. Other components of treatment	4a) Details of other interventions administered to the acupuncture group (e.g. moxibustion, cupping, herbs, exercises, lifestyle advice) - Advice for lifestyle habit included and no other interventions during the study allowed.
	4b) Setting and context of treatment, including instructions to practitioners, and information and explanations to patients - Participants will be informed that the acupuncture treatment is based on traditional Korean Medicine and previous studies on clinical trial.
5. Practitioner background	5) Description of participating acupuncturists (qualification or professional affiliation, years in acupuncture practice, other relevant experience) - Korean Medicine Doctors who have license and at least 2 years of experience of treating gastrointestinal diseases. They have studied acupuncture for more than 10 years and graduated university of Korean medicine. To ensure providing identical treatments, they finished 10 h of training and simulated the protocol.
6. Control or comparator interventions	6a) Rationale for the control or comparator in the context of the research question, with sources that justify this choice - No control intervention
	6b) Precise description of the control or comparator. If sham acupuncture or any other type of acupuncture-like control is used, provide details as for Items 1 to 3 above.

*LI* Large intestine meridian, *ST* Stomach meridian, *LR* Liver meridian, *PC* Pericardium meridian, *SP* Spleen meridian

### Outcome measurements

#### Primary outcome

##### Proportion of responder

Proportion of responder (PR) will be used to evaluate the proportion of patients who showed alleviation of FD symptoms, with relief of stomach pain or discomfort. PR is defined as the proportion of participants who answer “yes” to the adequate relief question more than half the time during the treatment period. The adequate relief question is, "After the last visit, have you had adequate relief of your stomach pain or discomfort?" The adequate relief question will be asked at every visit, starting with the second visit. PR will be calculated for the 4-week period. This indicator has been applied in several studies with functional intestinal diseases [9, 13, 14].

#### Secondary outcomes

##### Nepean dyspepsia index

The Nepean Dyspepsia Index (NDI) was validated by Talley et al. [15] and is a reliable index to measure the degree of dyspeptic symptoms, and the quality of life. Lee et al. developed the Korean version of the NDI [16]. Although, the NDI is composed of two categories, in this study, we will use only the symptom-based questions covering the period, severity, and degree of distress of 15 symptoms.

##### Short form 36-item health survey

The Short Form 36-item Health Survey (SF-36) is a self-report health status measure, that provides information about the overall health of the individual, and includes physical, mental, and social health [17]. The Korean version of SF-36 has undergone comprehensive psychometric evaluation of reliability and validity [18].

##### The state-trait anxiety inventory

The State-Trait Anxiety Inventory (STAI) [19] investigates anxiety as a psychological factor. The STAI is a questionnaire composed of the following 40 items evaluated using a 4-point Likert scale: 20 items evaluate the state of anxiety (anxiety triggered by a specific event) and 20 items identify the trait of anxiety (anxiety derived from personal characteristics). Higher scores indicate more severe anxiety. Anxiety has a significant correlation with dyspepsia [20, 21], and the effect of acupuncture on anxiety will be evaluated using the STAI.

##### Visual analog scale for dyspepsia

Participants will be requested to check the intensity of their abdominal discomfort on 0 to 100 numerical rating scale, with 0 representing “no discomfort” and 100 representing "the most intense discomfort he/she ever had".

##### Expectations for relief scale

This scale evaluates the degree of the patients’ expectations for the treatment. Subjects will be asked two questions, "How bothersome do you expect your dyspepsia to be at the end of treatment?" and "How much do you expect acupuncture treatment will relieve your clinical dyspepsia?" Participants will be asked to indicate their responses on a 0 to 100 numerical rating scale.

##### Massachusetts General Hospital acupuncture sensation scale

The descriptors in the “Subjective Acupuncture Sensation Scale” resulting in the Massachusetts General Hospital Acupuncture Sensation Scale (MASS) were expanded by Kong et al. [22]. The MASS includes soreness, aching, deep pressure, heaviness, fullness/distension, tingling, numbness, sharp pain, dull pain, warmth, cold, throbbing, and a blank row for subjects to describe their perceptions in their own words. Establishment of the MASS involved extensive review of the relevant literature and it is the most comprehensive assessment tool in measuring the needle sensations.

##### The consultation and relational empathy measure

The Consultation and Relational Empathy (CARE) measure was originally developed to assess empathy between doctor and patient. Mercer et al. [23] confirmed the reliability and validity. The CARE measure consists of 10 questions, and each question has six options from “poor” to “excellent” and “does not apply.”

##### Interpersonal reactivity index

Interpersonal Reactivity Index (IRI) investigates the degree of empathy defined as the "reactions of one individual to the observed experiences of another" [24]. The IRI consists of 28-items answered on a 5-point Likert scale, ranging from “Does not describe me well” to “Describes me very well.” The measure has four subscales, each containing seven different items. These subscales are: Perspective Taking, the tendency to spontaneously adopt the psychological point of view of others; Fantasy, taps the respondents’ tendencies to transpose themselves imaginatively into the feelings and actions of fictitious characters in books, movies, and plays; Empathic Concern, assesses “other-oriented” feelings of sympathy and concern for unfortunate others; and Personal Distress, measures “self-oriented” feelings of personal anxiety and unease intense interpersonal settings. Korean version of IRI was validated by Kang et al. [25].

##### Maximum tolerable volume

This outcome is recorded as part of the validated nutrient drink test [26]. It is designed to induce dyspepsia, by ingesting a standard meal. When compared to a barium meal radiograph study, it is simple, non-invasive assessment that has been widely used [27, 28]. After an overnight fast, participants will consume a canned drink (Ensure, 237 ml/can, Abbott Nutrition, Columbus, Ohio, USA), at a constant rate, until they reach the maximum level of fullness. At five-minute intervals, the subjects will verbally rate their overall fullness level, on a scale of 0 to 5 (0, threshold; 5, maximum satiety). Participants will be instructed to stop drinking when their fullness level reaches 5. The actual volume consumed is the maximum tolerable volume. Questionnaires for postprandial symptoms and intensity of abdominal discomfort will be administered 30 min after drinking. If participants cannot continue to consume the test drinks, for any reason, they can voluntarily stop at any time.

##### The pictorial representation of illness and self measure

The Pictorial Representation of Illness and Self Measure (PRISM) is a two dimensional pictorial method to assess the burden of suffering. The burden of suffering is defined as "a state of severe distress associated with events that threaten the intactness of the person." Patients illustrate their burden of suffering by the distance from “self” to an illness circle, where a shorter distance indicates a higher burden of suffering. The PRISM has been validated as a reliable method for assessing the burden of suffering in a variety of chronic diseases [29].

#### Participant timeline

The time schedule of enrollment, intervention, and assessments is summarized in Table 3.

**Table 3:** The schedule of enrollment, intervention, and assessments

Study period	Screening Visit	Acupuncture treatment period							
Visit		v1	v2	v3	v4	v5	v6	v7	v8
Week		1	1	2	2	3	3	4	4
Patients									
Informed Consent	√								
Demographics	√								
Physical examination	√								
History Examination	√								
Dyspepsia History Examination	√								
Inclusion/Exclusion Criteria	√								
Serologic test/ Evaluation	√								
BDI	√								
Randomization	√								
Trial evaluation									
Investigation of Concomitant medication		√^*^	√^*^	√^*^	√^*^	√^*^	√^*^	√^*^	√^*^
Adverse events		√^*^	√^*^	√^*^	√^*^	√^*^	√^*^	√^*^	√^*^
Outcomes									
AR of abdominal pain & discomfort			√^*^	√^*^	√^*^	√^*^	√^*^	√^*^	√^*^
VAS for dyspepsia	√	√^*^							√^†^
Expectations for Relief Scale		√^*^			√^*^				√^*^
MASS		√^†^	√^†^	√^†^	√^†^	√^†^	√^†^	√^†^	√^†^
The CARE measure		√^†^							√^†^
IRI		√^†^							√^†^
NDI		√^*^							√^†^
SF-36		√^*^							√^†^
STAI		√^*^							√^†^
PRISM		√^*^							√^†^
Nutrition drink test		√^*^							√^†^
Intervention									
Acupuncture treatment		√	√	√	√	√	√	√	√

^*^, conducted before acupuncture treatment; ^†^, conducted after acupuncture treatment; BDI, Beck Depression Inventory; *AR* Adequate Relief, *VAS* Visual analog scale, *MASS* Massachusetts General Hospital Acupuncture Sensation Scale, *CARE* Consultation and Relational Empathy, *IRI* Interpersonal Reactivty Index, *NDI* Nepean Dyspepsia Index, *SF-36* Short Form 36-item Health Survey, *STAI* State-Trait Anxiety Inventory, *PRISM* Pictorial Representation of Illness and Self Measure

### Sample size calculation

In the present study, we expected a 60% PR from the augmented group and up to a 30% PR from the limited group. These anticipated percentages were derived from previous studies [9, 14], and a consensus of experts in clinical gastroenterology. Significance was determined as α = 0.05, and a power of 1 – β = 0.80 was used. Accordingly, the required sample size in this trial was estimated according to the following formula where (p_a_ + p_l_)/2:

$$ n=\frac{{\left({\mathrm{Z}}_{\upalpha /2}\sqrt{2\overline{p}\left(12\overline{p}\right)}+ Z\beta \sqrt{p_a\left(1-{p}_a\right)+{p}_l\left(1-{p}_l\right)}\right)}^2}{{\left({p}_a-{p}_l\right)}^2} $$

Assuming p_a_ = 0.6 (p_a_: the effect on the augmented group) and p_l_ = 0.3 (p_l_: the effect on the limited group), a sample size of *n* = 40 is calculated to achieve 5% significance level and 80% power. Assuming a dropout rate of 10%, a total of 88 participants were needed with a 1:1 allocation to each group (44 participants per group).

### Statistical analysis

To compare demographic or clinical characteristics a two-sample *t*-test (in case of continuous data) or Chi-square test (in case of binary data) will be used. If a significant difference is identified between the augmented and limited groups, a general linear model-analysis of covariance will be used for continuous data, and logistic regression analysis will be used for binary data. To compare the mean value of two groups, a two-sample *t*-test will be used as a parametric method, and Mann-Whitney test will be used as a non-parametric method. The paired *t*-test will be used as parametric method and Wilcoxon signed ranks test will be used as non-parametric method to compare variables before and after intervention. The repeated measured *t*-test will be used to compare the mean value of variables between the two groups. A *P* value of <0.05 will be considered as statistical significance. Efficacy will be investigated as the difference between baseline (1 week) and the completion of treatment (4 weeks). To assess the efficacy of interventions, both intention to treat and per-protocol analysis will be performed. Participants who complete the clinical trial without any violations are targeted in per-protocol analysis. Statistical analysis other than described above will follow Guideline for Clinical Trial Statistics (Article No. 65615–13,553, December 29th 2000).

### Safety

Adverse events related to acupuncture treatment, including swelling, pain, bruising at the insertion site, discomfort, and dizziness will be assessed and recorded by Clinical Research Coordinator. Research physicians will manage adverse events, and the principle investigator will be notified. In the event of serious adverse events, the IRB and the sponsor will be notified within 24 h, regardless of causality. The principle investigator has the right terminate the study if serious adverse events occur.

### Quality control

To confirm the quality of the study, all practitioners of acupuncture will be required to have a license and more than 2 years of experience. To improve consistency, all researchers will be trained several times in both the augmented and limited context. Outcome measurements are retained in the source documents, the hospital or private records, and the laboratory results and records. Original data in source documents will be recorded into the electronic Case Report Form (e-CRF). The data entered into the e-CRF will be identical to the original data. Independent investigators and the Clinical Research Coordinator will verify the accuracy of the data in the e-CRF, and an independent statistician will analyze the data. If participants do not complete the study, the reasons will be fully documented.

### Monitoring

Monitoring will be carried out to assess whether the documents are accurate and precise in comparison with the reference, and that the study is performed in accordance with the approved protocol and regulations. A monitoring agent, designated by the sponsor, will be responsible for monitoring throughout the study. The principal investigator will provide source documents to monitoring agent. Monitoring includes review of e-CRF and informed consent forms.

## Discussion

This study will provide information about the influence of patient-practitioner interaction on the effect of acupuncture in functional dyspepsia, and increase the knowledge about the placebo effect on complementary and alternative medicine treatments in functional gastrointestinal diseases. This study will also compare the non-specific effects of the patient-practitioner relationship between Asian patients, with familiarity of acupuncture, and Western patients, who may not be familiar with acupuncture. We will investigate the participants’ experience and use of complementary and alternative medicine at the baseline. The results of this study will be published regardless of outcome in an article on accordance of STRICTA guidelines.

### Trial status

Recruitment began in February 2017.

## Abbreviations

CARE: Consultation and Relational Empathy
e-CRF: Electronic Case Report Form
FD: Functional dyspepsia
IRB: Institutional Review Board
IRI: Interpersonal Reactivity Index
LI: Large intestine meridian
LR: Liver meridian
MASS: The Massachusetts General Hospital Acupuncture Sensation Scale
NDI: The Nepean Dyspepsia Index
PC: Pericardium meridian
PR: Proportion of responder
PRISM: The Pictorial Representation of Illness and Self Measure
SF-36: The Short Form 36-item Health Survey
SP: Spleen meridian
ST: Stomach meridian
STAI: The State-Trait Anxiety Inventory
STRICTA: STandards for Reporting Interventions in Clinical Trials of Acupuncture
VAS: Visual analogue scale
